# Thermoresponsive Property of Poly(*N*,*N*-bis(2-methoxyethyl)acrylamide) and Its Copolymers with Water-Soluble Poly(*N*,*N*-disubstituted acrylamide) Prepared Using Hydrosilylation-Promoted Group Transfer Polymerization

**DOI:** 10.3390/polym15244681

**Published:** 2023-12-12

**Authors:** Xiangming Fu, Yanqiu Wang, Liang Xu, Atsushi Narumi, Shin-ichiro Sato, Xiaoran Yang, Xiande Shen, Toyoji Kakuchi

**Affiliations:** 1 Research Center for Polymer Materials, School of Materials Science and Engineering, Changchun University of Science and Technology, Weixing Road 7989, Changchun 130022, China; 18846321589@163.com (X.F.); yqwang@cust.edu.cn (Y.W.); m13069984921@163.com (L.X.); 18686350018@163.com (X.Y.); 2 Graduate School of Organic Materials Science, Yamagata University, 4-3-16 Jonan, Yonezawa 992-8510, Yamagata, Japan; narumi@yz.yamagata-u.ac.jp; 3 Division of Applied Chemistry, Faculty of Engineering, Hokkaido University, Sapporo 060-8628, Hokkaido, Japan; s-sato@eng.hokudai.ac.jp; 4 Chongqing Research Institute, Changchun University of Science and Technology, No. 618 Liangjiang Avenue, Longxing Town, Yubei District, Chongqing 401135, China

**Keywords:** poly(*N*,*N*-bis(2-methoxyethyl)acrylamide), thermoresponsive property, cloud point temperature, group transfer polymerization, hydrosilylation-promoted GTP

## Abstract

The group-transfer polymerization (GTP) of *N*,*N*-bis(2-methoxyethyl)acrylamide (MOEAm) initiated by Me_2_EtSiH in the hydrosilylation-promoted method and by silylketene acetal (SKA) in the conventional method proceeded in a controlled/living manner to provide poly(*N*,*N*-bis(2-methoxyethyl)acrylamide) (PMOEAm) and PMOEAm with the SKA residue at the α-chain end (MCIP-PMOEAm), respectively. PMOEAm-*b*-poly(*N,N-*dimethylacrylamide) (PDMAm) and PMOEAm-*s*-PDMAm and PMOEAm-*b*-poly(*N,N*-bis(2-ethoxyethyl)acrylamide) (PEOEAm) and PMOEAm-*s*-PEOEAm were synthesized by the block and random group-transfer copolymerization of MOEAm and *N,N-*dimethylacrylamide or *N,N*-bis(2-ethoxyethyl)acrylamide. The homo- and copolymer structures affected the thermoresponsive properties; the cloud point temperature (*T*_cp_) increasing by decreasing the degree of polymerization (x). The chain-end group in PMOEAm affected the *T*_cp_ with PMOEAm_x_ > MCIP-PMOEAm_x_. The *T*_cp_ of statistical copolymers was higher than that of block copolymers, with PMOEAm_x_-*s*-PDMAm_y_ > PMOEAm_x_-*b*-PDMAm_y_ and PMOEAm_x_-*s*-PEOEAm_y_ > PMOEAm_x_-*b*-PEOEAm_y_.

## 1. Introduction

Thermoresponsive polymers exhibit a reversible phase transition in response to a change in temperature. The most common thermoresponsive polymers are those with lower critical solution temperatures, which are soluble in a particular solvent at low temperatures but insoluble at high temperatures [1,2,3,4,5], such as poly(*N*-isopropylacrylamide) (PNIPAm) [6,7,8,9], poly(ethylene glycol) [10,11,12], poly(2-hydroxyethyl methacrylate) [13,14,15], poly(methacrylic acid) [16,17,18], poly(styrene sulfonate) [19,20], poly(vinyl alcohol) [21,22,23], poly(*N*-vinylcaprolactam) [24,25,26], and poly(*N*,*N*-diethylacrylamide) (PDEAm) [27,28]. For example, PNIPAm is soluble in water at room temperature but becomes insoluble in water at body temperature, which renders it suitable for drug delivery, tissue engineering, biosensing, self-assembly, and other uses [29]. Furthermore, the sol-gel conversion of polyacrylamide in water is widely used in applications such as drilling, acidification, water purification, and flocculation [30].

Thus, the development of new thermoresponsive polymers is important to realize new applications. In polymer design, we need to consider that the thermoresponsive property is closely related to the polymer structure, including the primary structure of the polymer main and side chains [31], polymer stereoregularity [32], molecular mass and polydispersity [33], polymer end groups [34], and type of structure, i.e., block [35,36,37,38], graft [39,40,41], cyclic hyperbranched [42,43], and star-shaped [44,45]. To precisely synthesize these polymers, living radical polymerization methods [46], such as nitroxide-mediated polymerization [47,48], atom transfer radical polymerization [49,50,51], and reversible addition/fragmentation chain transfer (RAFT) polymerization [52,53,54], are widely used. In these polymerization methods, end groups derived from polymerization initiators are inevitably introduced into the resulting polymers. Since the end groups of polymers affect the thermoresponsive properties, these synthetic methods are not suitable for the characterization of new thermoresponsive polymers in terms of, for example, the molecular mass dependence of the thermoresponsive properties.

Group-transfer polymerization (GTP) is a reliable living polymerization method for polar monomers such as (meth)acrylate, which can be performed using conventional catalysts to produce well-defined polymers [55]. For instance, Taton et al., Waymouth et al., and our group reported that organocatalysts could effectively promote the controlled/living GTP of (meth)acrylate [56,57], alkyl sorbate [58], alkyl crotonate [59], methacrylonitrile [60], and *N*,*N*-disubstituted acrylamide (DSAm) [61]. These conventional GTP systems require the use of silyl ketene acetal (SKA) or silyl ketene aminal (SKAm) as an initiator, resulting in the attachment of residual groups derived from SKA or SKAm at the α-chain end of the obtained polymers. Recently, we developed a new GTP method that does not require adding SKA or SKAm beforehand, i.e., the polymerization of (meth)acrylate and acrylamide monomers with hydrosilane (R_3_SiH) using tris(pentafluorophenyl)borane (B(C_6_F_5_)_3_) as the catalyst, which proceeded through a controlled/living GTP mechanism to produce well-defined polymers without α-chain-end groups derived from SKA or SKAm [62,63]. In this GTP method, SKA or SKAm was formed via the B(C_6_F_5_)_3_-catalyzed hydrosilylation of a monomer and R_3_SiH in the polymerization system prior to the polymerization. We applied the hydrosilylation-promoted GTP method to the synthesis of thermoresponsive polymers capped with hydrogen atoms in both chain ends, such as PDEAm and poly(*N*-methyl,*N*-*n*-propylacrylamide) [64].

**Scheme 1:**
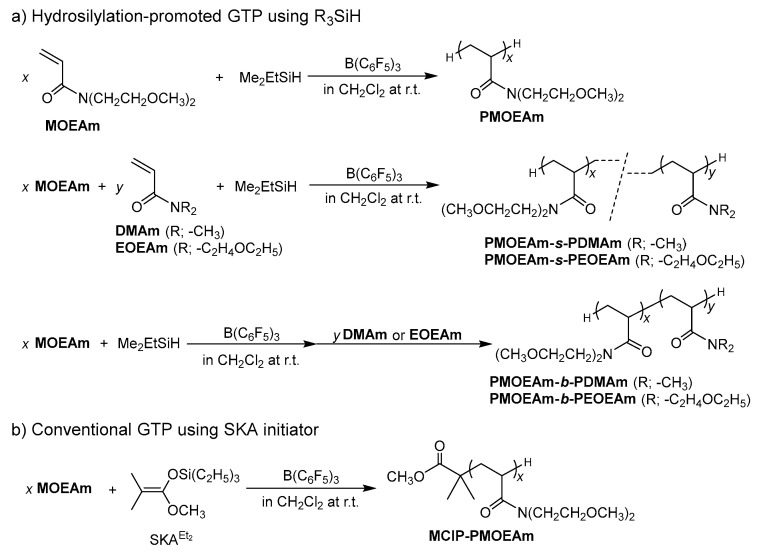
(**a**) Synthesis of PMOEAm, PMOEAm-*b*-PDMAm and PMOEAm-*s*-PDMAm, and PMOEAm-*b*-PEOEAm and PMOEAm-*s*-PEOEAm via hydrosilylation-promoted group-transfer polymerization (GTP) and (**b**) synthesis of PMOEAm functionalized with a (methoxycarbonyl)isopropyl (MCIP) group at the α-chain end (MCIP-PMOEAm) via conventional GTP using silylketene acetal (SKA) as an initiator.

Poly(*N*,*N*-bis(2-methoxyethyl)acrylamide) (PMOEAm) has been synthesized using radical polymerization methods as a thermoresponsive poly(*N*,*N*-disubstituted acrylamide). For example, conventional radical polymerization was used for the preparation of PMOEAm [65], PMOEAm-*s*-PNIPAm [66,67], and carboxy-terminated PMOEAm [68], while RAFT polymerization was adopted to produce PMOEAm [69] and PMOEAm-*b*-poly(*N*,*N*-dimethylacrylamide) (PDMAm) [70], whose α,ω-ends were capped with residues derived from the RAFT agent. In addition, we reported that the conventional GTP of *N*,*N*-bis(2-methoxyethyl)acrylamide (MOEAm) with SKA using an organocatalyst produced PMOEAm functionalized with the SKA residue at the α-chain end ((methoxycarbonyl)isopropyl (MCIP)-PMOEAm, Scheme 1b). Thus, toward the design and synthesis of thermoresponsive materials consisting of PMOEAm, clarifying the thermoresponsive property of PMOEAm without the influence of terminal groups is important. Here, we report the synthesis of PMOEAm capped with hydrogen atoms at both chain ends via the hydrosilylation-promoted GTP of MOEAm with dimethylethylsilane (Me_2_EtSiH) using B(C_6_F_5_)_3_ as a catalyst. To broaden the thermoresponsive range of PMOEAm, we synthesized statistical and block copolymers of PMOEAm with water-soluble PDMAm (nonthermoresponsive), and thermoresponsive poly(N,N-bis(2-ethoxyethyl)acrylamide) (PEOEAm), utilizing hydrosilylation-promoted group-transfer copolymerization (GTcoP), as displayed in Scheme 1a [71] (Scheme 1a). The effects of differences in the polymer structures between PMOEAm and MCIP-PMOEAm, PMOEAm-*b*-PDMAm and PMOEAm-*s*-PDMAm, and PMOEAm-*b*-PEOEAm and PMOEAm-*s*-PEOEAm on the thermoresponsive behavior were investigated by measuring the nuclear magnetic resonance (NMR) spectra and dynamic light scattering (DLS) of these polymer solutions before and after the cloud point temperature (*T*_cp_).

## 2. Materials and Methods

### 2.1. Materials

Dichloromethane (CH_2_Cl_2_, >99.5%; water content < 0.001%), methyl alcohol (MeOH), and calcium hydride (CaH_2_) were purchased from Kanto Chemicals Co., Inc. (Tokyo, Japan). Bis(2-methoxyethyl)amine, DMAm, and Me_2_EtSiH were obtained from Tokyo Kasei Kogyo Co., Ltd. (Tokyo, Japan). B(C_6_F_5_)_3_ was procured from Wako Pure Chemical Industries, Ltd. (Tokyo, Japan) and utilized after recrystallization from n-hexane at −30 °C. DMAm and CH_2_Cl_2_ were distilled using CaH_2_ and degassed through three freeze-pump-thaw cycles before being stored under an Ar atmosphere for future use. All other chemicals were purchased from suppliers and used without purification. Polymerizations were carried out in a glove box under Ar atmosphere at 25 °C.

### 2.2. Measurements

^1^H NMR spectra were obtained using a Bruker Avance III HD 500 spectrometer from Bruker Corporation (Billerica, MA, USA). Polymerization solutions were prepared in a Mikrouna glove box with a gas purification system consisting of molecular sieves and a copper catalyst and were filled with a dry Ar atmosphere (with H_2_O and O_2_ contents of less than 1 ppm). The water and oxygen levels inside the glove box were monitored using the MK-XTR-100 and MK-OX-SEN-1 sensors from Mikarona Industrial Intelligent Technology Co., Ltd. (Shanghai, China), respectively. The polymers’ number-average molecular mass (M_n,SEC_) and polydispersity index (Đ) were measured through size exclusion chromatography (SEC) at 40 °C. The Agilent high-performance liquid chromatography system (1260 Infinity II) was utilized in *N*,*N*-dimethylformamide (DMF) containing 0.01% lithium chloride. A solution of 1.0 mol L^−1^ was flowed through Agilent Polar Gel-M columns (exclusion limit, 2 × 10^4^ g mol^−1^) and Polar Gel-M columns (exclusion limit, 4 × 10^6^ g mol^−1^) (7.5 × 300 mm; average bead size, 5 μm) (Agilent Technologies Inc. Shanghai, China) at a rate of 1.0 mL min^−1^. Cloud-point measurements were taken on a Jasco V-770 ultraviolet–visible (UV–vis) spectrophotometer (Tokyo, Japan), which was equipped with a Jasco CTU-100 temperature controller. The temperature was then increased at a rate of 1 °C min^−1^ while the path length was 10 mm. Changes in transmission were recorded at 500 nm with varying temperatures. The hydrodynamic radius (*R*_h_) of the produced polymers was analyzed through a Dyna Pro Nanostar^®^ instrument from Wyatt Technology in Santa Barbara, CA, USA.

### 2.3. Synthesis of PMOEAm

A solution of MOEAm (749.0 mg, 4 mmol), Me_2_EtSiH (5.28 µL, 40 µmol), and B(C_6_F_5_)_3_ (2.1 mg, 4.0 µmol) in CH_2_Cl_2_ (3.96 mL) was stirred for 12 h at room temperature in a glove box under an Ar atmosphere. To terminate the polymerization, a small amount of MeOH was added, and then the crude product was purified by dialysis against acetone to obtain a white solid product. The yield was 408.7 mg (54.6%).

### 2.4. Synthesis of MCIP-PMOEAm

A solution of MOEAm (749.0 mg, 4 mmol), 1-methoxy-1-(triethylsiloxy)-2-methyl-1-propene (SKA^Et^; 8.64 mg, 40 µmol), and B(C_6_F_5_)_3_ (2.1 mg, 4.0 µmol) in CH_2_Cl_2_ (3.96 mL) was stirred for 8 h at room temperature in a glove box under an Ar atmosphere. To terminate the polymerization, a small amount of MeOH was added, and then the crude product was purified by dialysis against acetone to obtain a white solid product. The yield was 408.7 mg (55%).

### 2.5. Synthesis of PMOEAm-s-PDMAm

A solution containing MOEAm (375.5 mg, 2 mmol), DMAm (198.4 mg, 2 mmol), Me_2_EtSiH (5.28 µL, 40 µmol), and B(C_6_F_5_)_3_ (2.1 mg, 4.0 µmol) in CH_2_Cl_2_ (3.96 mL) was stirred for 12 h at room temperature in a glove box under an Ar atmosphere. To terminate the polymerization, a small amount of MeOH was added, and the crude product was then purified by dialysis against acetone to obtain a white solid product. The yield was 369.1 mg (50%).

### 2.6. Synthesis of PMOEAm-b-PDMAm

A solution containing MOEAm (375.5 mg, 2 mmol), Me_2_EtSiH (5.28 µL, 40 µmol), and B(C_6_F_5_)_3_ (2.1 mg, 4.0 µmol) in CH_2_Cl_2_ (3.96 mL) was stirred under an Ar atmosphere at room temperature in a glove box for 12 h. A sample of the reaction solution was taken to confirm the complete MOEAm consumption via ^1^H NMR spectroscopy. Afterward, DMAm (198.4 mg, 2 mmol) was introduced to the polymerization mixture and stirred for 36 h. The crude product was then purified following the same procedure used in the synthesis of PEOEAm. The result was a white solid polymer with a yield of 345.9 mg (47%).

## 3. Results and Discussion

### 3.1. Synthesis of PMOEAm

**Figure 1:**
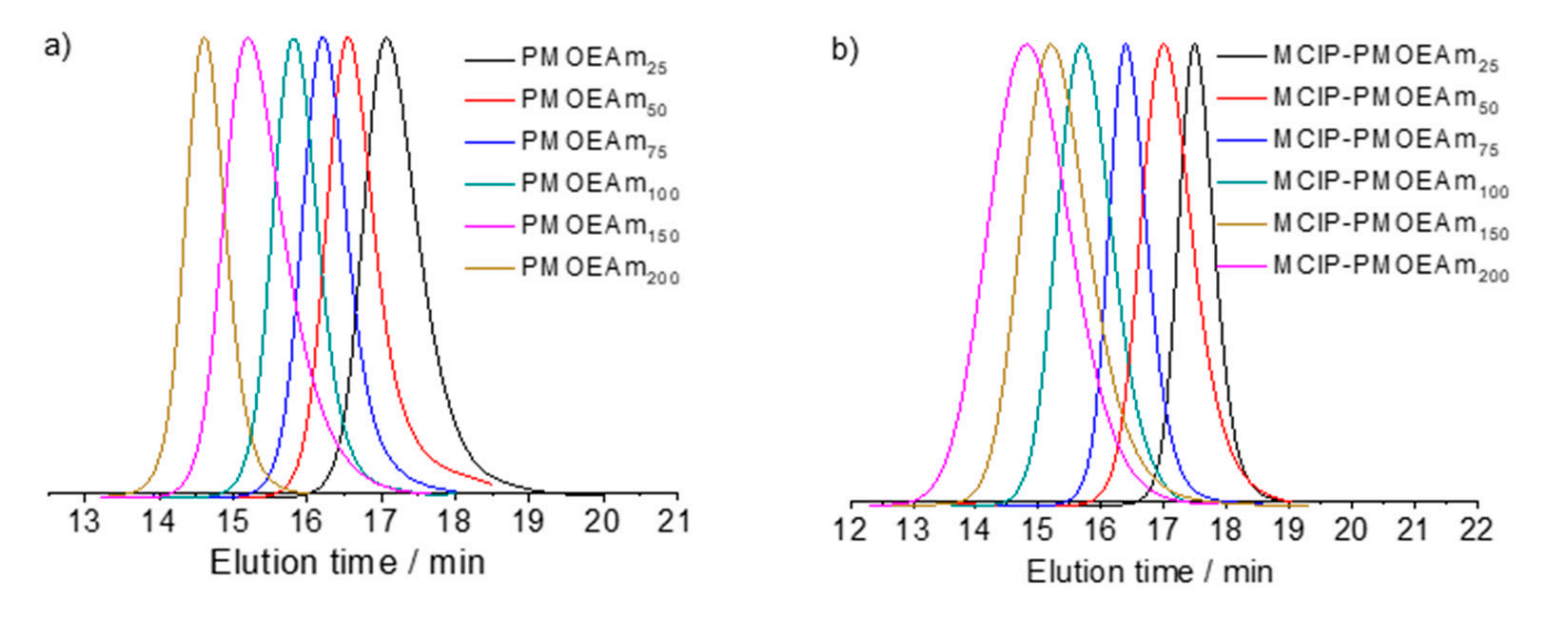
SEC traces of (**a**) PMOEAm and (**b**) MCIP-PMOEAm (eluent, DMF containing lithium chloride (0.01 mol L^−1^); flow rate, 1.0 mL min^−1^).

We reported preliminary results for the GTP of MOEAm with SKA^Et^ or Me_2_EtSiH using B(C_6_F_5_)_3_ as the catalyst, i.e., the conventional and hydrosilylation-promoted methods, respectively [72]. In order to clarify the effect of the chain end group, it is necessary to use polymers synthesized under similar polymerization conditions for both GTPs. For the hydrosilylation-promoted GTP of MOEAm with Me_2_EtSiH, the polymerization was performed at a [MOEAm]_0_/[Me_2_EtSiH]_0_/[B(C_6_F_5_)_3_]_0_ ratio of 25/1/0.1. The *M*_n,SEC_ of the obtained polymer was 4.5 kg mol^−1^, which was in good agreement with the calculated molecular mass (*M*_n,calcd_) of 4.7 kg mol^−1^ (Table 1, run 1). The polydispersity index (Đ) of the obtained polymer was as low as 1.13 (Figure 1a). Similarly, the conventional GTP of MOEAm was performed at a [MOEAm]_0_/[SKA^Et^]_0_/[B(C_6_F_5_)_3_]_0_ ratio of 25/1/0.1 to obtain a polymer with a *M*_n,SEC_ of 5.1 kg mol^−1^, which was close to the *M*_n,calcd_ of 4.9 kg mol^−1^ (Table S1). The SEC trace of the resulting polymer was unimodal with a low Đ of 1.11 (Figure 1b).

**Figure 2:**
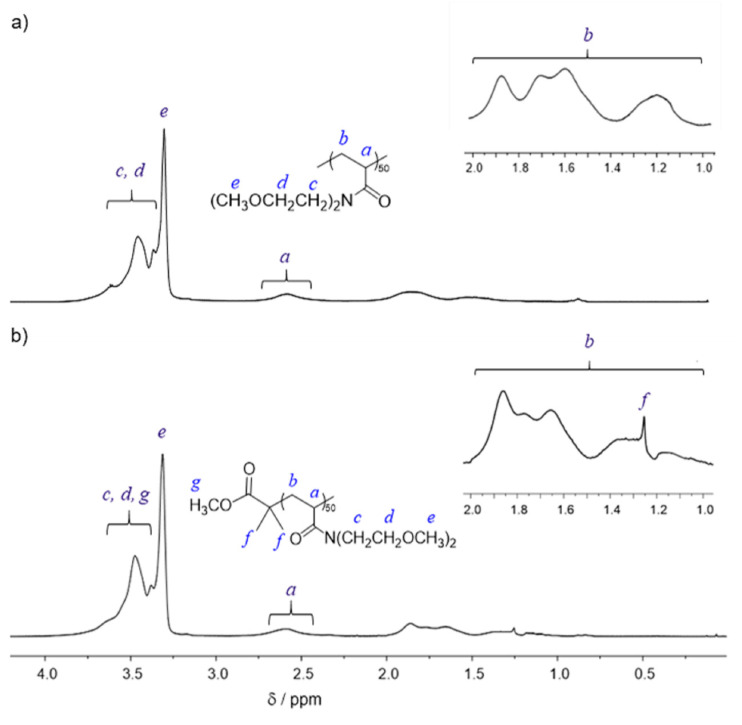
^1^H NMR spectra of (**a**) PMOEAm_25_ and (**b**) MCIP-PMOEAm_25_ in CDCl_3_.

The ^1^H NMR spectra of the polymers obtained using the two initiation methods were almost identical, showing signals due to the –NCH_2_CH_2_O–, –OCH_3_, –CH_2_CH–, and –CH_2_CH– groups at 3.83–3.88, 3.31–3.38, 0.99–2.01, and 2.45–2.75 ppm, respectively. In addition, signals due to the –C(CH_3_)_2_ group as a residue of SKA^Et^ were observed at 1.26 ppm for the polymer prepared using the conventional initiation method (Figure 2). More detailed information on the resulting polymer structures was obtained via matrix-assisted laser desorption/ionization time-of-flight mass spectrometry (MALDI-TOF MS) measurements.

**Figure 3:**
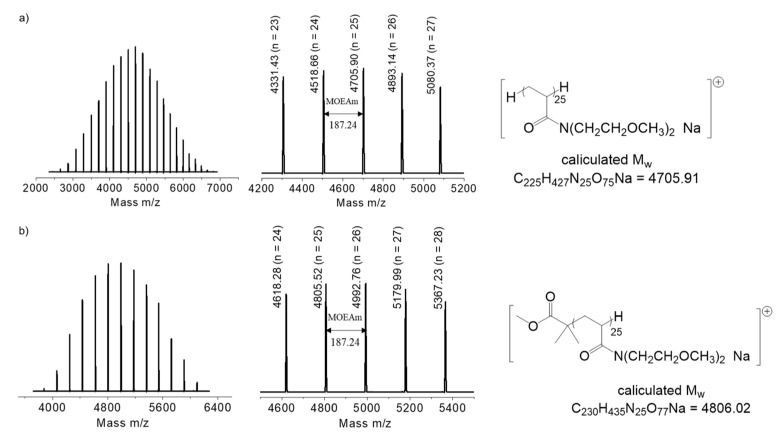
MALDI-TOF MAS spectra of (**a**) PMOEAm_25_ with a *M*_n,SEC_ of 4.5 kg mol^−1^ and a Đ of 1.13 (Table 1, run 1) and (**b**) MCIP-PMOEAm_25_ with a M_n,SEC_ of 5.1 kg mol^−1^ and a Đ of 1.11 (Table S1, run 1).

The MALDI-TOF MS spectrum depicted in Figure 3a reveals a sole set of molecular ion peaks with an adjacent peak distance of 187.24 Da, consistent with the molecular mass prediction of MOEAm as the constitutional repeat unit. Moreover, the *m*/*z* values of each molecular ion peak were in accordance with the sodium-cationized polymer composition of [H-MOEAmn-H + Na]^+^ (molecular formula: C_9n_H_17n_ + _2_H_2_NnO_3n_Na). For example, an *m*/*z* value of 4705.82 Da corresponds to a sodium-cationized 25-unit polymer structure of [H-MOEAm_25_-H + Na]^+^, with a theoretical monoisotopic value of 4705.03 Da for the molecular formula C_225_H_427_N_25_O_75_Na.

Similarly, for the polymer obtained using the conventional GTP with SKA^Et^, only one series of molecular ion peaks is observed in Figure 3b, and the difference in the *m/z* values between each peak is consistent with the molecular mass predicted for MOEAm. In addition, the *m/z* value of each molecular ion peak can be attributed to the sodium-cationized polymer with the MCIP group, the desilylated SKA^Et^, at the α-chain end and a hydrogen atom at the ω-chain end of [CH_3_O_2_CC(CH_3_)_2_-MOEAm*_n_*-H + Na]^+^ (C_9n+5_H_17n+9_N_n_O_3n+2_Na). For example, an *m/z* of 4805.52 Da for a specific peak corresponds to a [CH_3_O_2_CC(CH_3_)_2_-MOEAm_25_-H + Na]^+^ with a theoretical monoisotopic value of 4806.09 for C_230_H_436_N_25_O_77_Na. In the conventional and hydrosilylation-promoted GTP reactions, the resulting polymers consisted only of monomeric units, although the difference between Me_2_EtSiH and SKA^Et^ used in the initiation reaction was reflected in the structure of the initiating end of the polymer, i.e., PMOEAm_25_ and CH_3_O_2_CC(CH_3_)_2_-MOEAm_25_, respectively.

**Figure 4:**
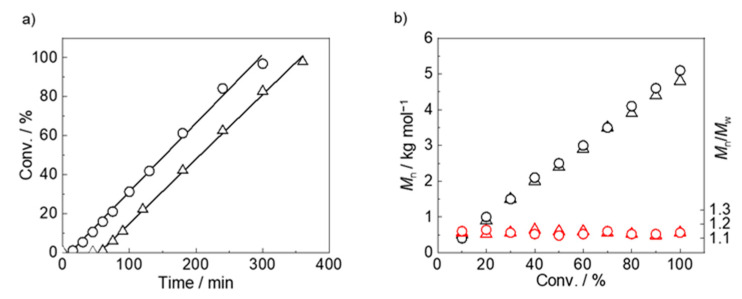
(**a**) Zero-order kinetic plots and (**b**) dependence of molar mass (*M*_n_) and polydispersity index (Đ) on monomer conversion (Conv.) in the B(C_6_F_5_)_3_-ctalyzed GTP of MOEAm with (○) Me_2_EtSiH and (Δ) SKA^Et^ ([MOEAm]_0_/[Me_2_EtSiH or SKA^Et^]_0_/[B(C_6_F_5_)_3_]_0_, 100/1/0.1; [MOEAm]_0_, 1.0 mol L^−1^).

Furthermore, the polymerization features of the GTP of MOEAm initiated using two different methods were compared by evaluating the polymerization kinetics. The GTP of MOEAm was performed at a [MOEAm]_0_/[Me_2_EtSiH or SKA^Et^]_0_/[B(C_6_F_5_)_3_]_0_ ratio of 100/1/0.1 and a [MOEAm]_0_ of 1.0 mol L^−1^ in CH_2_Cl_2_ at 25 °C. Although Et_3_SiH should be used for an accurate comparison with SKA^Et^, the hydrosilylation-promoted GTP of DSAm with Et_3_SiH did not proceed in a controlled/living manner; therefore, Me_2_EtSiH was used instead of Et_3_SiH. As shown in Figure 4, both GTP systems exhibited induction times (*t*_i_), with the *t*_i_ for Me_2_EtSiH (13.1 min) being smaller than that for SKA^Et^ (55.0 min) due to the difference in both initiation reactions. In the kinetic plot of MOEAm with Me_2_EtSiH and SKA^Et^, a clear zero-order relationship between polymerization time and monomer conversion was observed, with *M*_n_,_SEC_ increasing linearly with increasing monomer conversion and Đ remaining at low values. The polymerization rates of both GTP reactions of MOEAm were almost the same with *k*_p,obs_ values of 0.35 min^−1^ for Me_2_EtSiH and 0.33 min^−1^ for SKA^Et^. These results confirmed that the difference in the initiation method affected only the induction time in the early stages of the polymerization but had no effect on the propagation rate.

### 3.2. Synthesis of Block and Statistical Copolymers of PMOEAm

The response performance of thermoresponsive polymers is controlled by adjusting the degree of polymerization and introducing polymer chain-end groups. The use of block and statistical copolymer systems based on thermoresponsive polymers is an additional approach to controlling the thermoresponsive property. In this study, DMAm as a simple DSAm and EOEAm as an analog of MOEAm were used as comonomers. The block GTcoP reactions of MOEAm and DMAm or EOEAm with Me_2_EtSiH using B(C_6_F_5_)_3_ as the catalyst were performed using a sequential monomer addition method by varying the molar ratio of [MOEAm]_0_ and [DMAm or EOEAm]_0_. The copolymerization results are listed in Table 2 and Table 3, respectively.

In the hydrosilylation-promoted GTcoP of MOEAm and DMAm with a [MOEAm]_0_/[DMAm]_0_/[Me_2_EtSiH]_0_ molar ratio of 50/50/1 (Table 2, run 9), after confirming the quantitative consumption of MOEAm in the first GTP, DMAm was added to the living PMOEAm system to perform the second GTP. The progress of GTcoP was verified through the shift in the SEC traces of the resulting polymers between the first and second GTPs while maintaining a Đ value below 1.13, as illustrated in Figure S1. The *M*_n,SEC_ of the resulting polymer increased from 9.6 kg mol^−1^ at the first GTP to 14.4 kg mol^−1^ at the second GTP, consistent with the *M*_n,calcd_ values of 9.4 and 14.3 kg mol^−1^, respectively. Furthermore, the block GTcoP of MOEAm and EOEAm performed with a [MOEAm]_0_/[DMAm]_0_/[Me_2_EtSiH]_0_ molar ratio of 50/50/1 gave similar results to the block GTcoP of MOEAm and DMAm; the *M*_n,SEC_ and Đ values of the resulting polymers were 9.1 kg mol^−1^ and 1.12 for the first GTP and 20.2 kg mol^−1^ and 1.11 for the second GTP. Note that the *M*_n,SEC_ values were close to the *M*_n,calcd_ values of 9.4 and 20.1 kg mol^−1^, respectively. In the ^1^H NMR spectrum of the resulting copolymer (Figure S3a), signals due to the –NCH_2_CH_2_O– and –OCH_3_ groups were observed at 3.39–3.87 and 3.31–3.39 ppm, respectively, and a signal appearing at 2.79–3.22 ppm can be attributed to the –NCH_3_ group of the MOEAm and DMAm units incorporated in the polymer. Similarly, signals due to the –OCH_3_ group of PMOEAm and the –OCH_2_CH_3_ group of PEOEAm were observed at 3.31–3.39 and 1.12–1.23 ppm, respectively (Figure S4a), which correspond to the MOEAm and EOEAm units incorporated in the copolymer. These results support the copolymer structures of PMOEAm_50_-*b*-PDMAm_50_ and PMOEAm_50_-*b*-PEOEAm_50_. The polymerization results of the synthesis of PMOEAm_x_-*b*-PDMAm_y_ and PMOEAm_x_-*b*-PEOEAm_y_ with other x/y ratios are summarized in Table 2.

Statistical copolymers of PMOEAm-*s*-PDMAm and PMOEAm-*s*-PEOEAm were prepared via the hydrosilation-promoted GTcoP of MOEAm and DMAm or EOEAm, respectively. Table 3 lists the copolymerization results. For example, random GTcoP reactions were performed with a [MOEAm + DMAm or EOEAm]_0_/[Me_2_EtSiH]_0_ molar ratio of (50 + 50)/1 (runs 21 and 26, respectively). The *M*_n,SEC_ and Đ values of the resulting polymers were 14.5 kg mol^−1^ and 1.14 (run 21) and 20.5 kg mol^−1^ and 1.13 (run 26), with the *M*_n,SEC_ values agreeing with the *M*_n,calcd_ of 14.3 and 20.1 kg mol^−1^, respectively. The ^1^H NMR spectra of both polymers with an x/y ratio of 50/50 were essentially identical to those of PMOEAm_50_-*b*-PDMAm_50_ and PMOEAm_50_-*b*-PEOEAm_50_ (Figures S3b and S4b, respectively). Similar to PMOEAm_50_-*s*-PDMAm_50_ and PMOEAm_50_-*s*-PEOEAm_50_, other statistical copolymers with different x/y ratios were synthesized via the corresponding random GTcoP; the *M*_n,SEC_ values of the obtained copolymers were consistent with the *M*_n,calcd_ values, and the SEC traces were unimodal with low Đ values (Figure S2).

The thermal phase transition behavior of a copolymer depends on the type of monomer sequence, i.e., alternating, block, or random sequence, which can be estimated by the monomer reactivity ratio (*r*). Therefore, the *r* values of the random GTcoP of MOEAm (*r*_MOEAm_) and DMAm (*r*_DMAm_) and those of MOEAm (*r*_MOEAm_) and EOEAm (*r*_EOEAm_) were determined using the Kelen–Tüdös method (see Supporting Information). The obtained *r*_MOEAm_ and *r*_DMAm_ values of 0.39 and 0.94, respectively, indicate that the sequence of the two monomers is relatively alternating-rich, whereas the *r*_MOEAm_ of 1.45 and the *r*_EOEAm_ of 1.03 suggest that the sequence of the two monomers is slightly block-rich. Furthermore, the number-average sequence length of the MOEAm unit (*l*_MOEAm_) determined using the *r* values reflects the isolation tendency of the MOEAm–MOEAm diad. When x increased from 30 to 90 (x + y = 100) for PMOEAm_x_-*s*-PDMAm_y_ and PMOEAm_x_-*s*-PEOEAm_y_, *l*_MOEAm_ increased from 1.17 to 2.56, and *l*_DMAm_ decreased from 3.19 to 1.27, while *l*_MOEAm_ increased from 1.62 to 6.80 and *l*_DMAm_ decreased from 3.40 to 1.26. These results indicate that the random GTcoP of MOEAm and DMAm or EOEAm produced PMOEAm-*s*-PDMAm with an alternating-rich sequence of both monomer units and PMOEAm-*s*-PEOEAm with a block-rich sequence of both monomer units, respectively.

### 3.3. Thermoresponsive Property of PMOEAm and Its Copolymers

**Figure 5:**
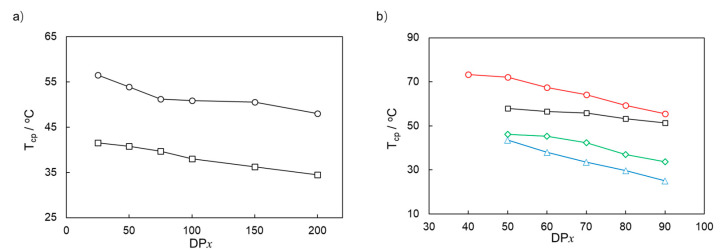
Dependence of *T*_cp_ on DP*_x_* for (**a**) (○) PMOEAm*_x_* and (□) MCIP-PMOEAm*_x_* and (**b**) (○) PMOEAm*_x_*-*s*-PDMAm*_y_*, (□) PMOEAm*_x_*-*b*-PDMAm*_y_*, (◊) PMOEAm*_x_*-*s*-PEOEAm*_y_*, and (∆) PMOEAm*_x_*-*b*-PEOEAm*_y_*.

The thermal phase transition behavior of PMOEAm and its copolymers were assessed at *T*_cp_, which is the point where the transmittance attains 50% of the transmittance-temperature curve plotted by monitoring an aqueous polymer solution (10 g L^−1^) using UV-vis spectroscopy at a wavelength of 500 nm. Figure S7 illustrates the findings. The thermoresponsive property of PMOEAm was compared with that of PMOEAm with the SKA residue at the α-chain end and block and statistical copolymers of PMOEAm and PDMAm or PEOEAm to investigate the effect of the chain-end group of the homopolymer and the monomer sequence of block and statistical copolymers on the thermoresponsive property. The dependence of *T*_cp_ on the degree of polymerization (DP_x_) for PMOEAm_x_ and MCIP-PMOEAm_x_ is shown in Figure 5a. The *T*_cp_ of both polymers decreased as DP*_x_* increased, from 56.5 °C to 48.0 °C for PMOEAm*_x_* and from 41.5 °C to 34.5 °C for MCIP-PMOEAmx. The relationship between DP_x_ and *T*_cp_ for both polymers was very similar, but the *T*_cp_ of MCIP-PMOEAm_x_ was, on average, 13.4 °C lower than that of PMOEAm_x_, regardless of the DP_x_. These results indicate that the –C(CH_3_)_2_ group acted as a hydrophobic group, reducing the *T*_cp_ of PMOEAm_x_. Therefore, a polymer without substituents at the chain ends should be used to determine whether the polymer exhibits thermoresponsive properties. The thermal phase transition should be accompanied by a change in the aggregate size of the copolymers, which was confirmed by measuring their hydrodynamic radius (*R*_h_) in water before and after the *T*_cp_. The *R*_h_ values increased drastically from 15.2 nm at 20 °C to 425.6 nm at 60 °C for the PMOEAm_50_ polymer with a *T*_cp_ of 53.9 °C and from 16.1 nm at 20 °C to 457.2 nm at 60 °C for the MCIP-PMOEAm_50_ polymer with a *T*_cp_ of 40.8 °C (Table 4).

**Figure 6:**
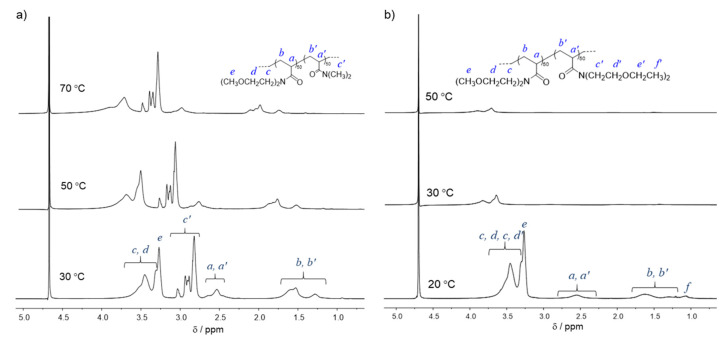
^1^H NMR spectra of (**a**) PMOEAm_50_-*b*-PDMAm_50_ measured in D_2_O at 30, 50, and 70 °C and (**b**) PMOEAm_50_-*b*-PEOEAm_50_ measured in D_2_O at 20, 30, and 50 °C.

For all copolymer systems, the *T*_cp_ increased with a decreasing DP_x_ from 51.4 °C to 58.0 °C for PMOEAm_x_-*b*-PDMAm_y_, from 55.5 °C to 73.4 °C for PMOEAm_x_-*s*-PDMAm_y_, from 25.0 °C to 43.5 °C for PMOEAm_x_-*b*-PEOEAm_y_, and from 33.7 °C to 46.2 °C for PMOEAm_x_-*s*-PEOEAm_y_ (Figure 5 and Figure 6b). PDMAm is nonthermoresponsive and highly water soluble, whereas PEOEAm is thermoresponsive with a low *T*_cp_ and less water soluble. This is reflected in the *T*_cp_ of the block and statistical copolymers of PMOEAm-*b*-PDMAm and PMOEAm_x_-*s*-PDMAm, which were higher than those of PMOEAm-*b*-PEOEAm and PMOEAm-*s*-PEOEAm. For PMOEAm and its block and statistical copolymers, the *T*_cp_ increased slightly from 53.9 °C for PMOEAm_50_ to 58.0 °C for PMOEAm_50_-*b*-PDMAm_50_ and increased considerably to 72.1 °C for PMOEAm_50_-*s*-PDMAm_50_ due to the alternating-rich monomer sequence. Meanwhile, the *T*_cp_ decreased from 53.9 °C for PMOEAm_50_ to 43.5 °C for PMOEAm_50_-*b*-PDMAm_50_ and increased slightly to 46.2 °C for PMOEAm_50_-*s*-PEOEAm_50_. The slight difference in *T*_cp_ between the block and statistical copolymers can be attributed to the lower degree of blockiness in the monomer sequence in the statistical copolymer.

The phase transition behavior was confirmed by measuring ^1^H NMR spectra at different temperatures. In the ^1^H NMR spectra of PMOEAm_50_-*b*-PDMAm_50_ with a *T*_cp_ of 58.0 °C, signals attributed to the –OCH_3_ group of PMOEAm and the –NCH_3_ group of PDMAm were observed at 3.19–3.30 and 2.73–3.07 ppm, respectively, at 30 °C. These signals shifted to a low magnetic field, and the intensity of the signal of the –OCH_3_ group decreased compared with that of the –NCH_3_ group at 50 °C. The signal of the –OCH_3_ group completely disappeared at 75 °C. A similar thermal phase transition was observed for PMOEAm_50_-*s*-PDMAm_50_ with a *T*_cp_ of 72.1 °C (Figure S5). Furthermore, an increase in *R*_h_ from 16.7 nm at 20 °C to 402.8 nm at 70 °C for PMOEAm_50_-*b*-PDMAm_50_ and from 14.5 nm at 20 °C to 349.9 nm at 75 °C for PMOEAm_50_-*s*-PDMAm_50_ was observed. These results indicate that PMOEAm and PDMAm acted as hydrophobic and hydrophilic moieties, respectively, resulting in the formation of aggregates comprising a PMOEAm core and a PDMAm shell. In the ^1^H NMR spectra in the D_2_O of PMOEAm_50_-*b*-PEOEAm_50_ with a *T*_cp_ of 25.0 °C (Figure 5b), the signal at 1.11 ppm attributed to the –OCH_2_CH_3_ groups of PEOEAm decreased at 20 °C and disappeared completely at 30 °C, which may be partly due to the low *T*_cp_ of 13.9 °C for the PEOEAm_50_ moiety. A signal due to the –OCH_3_ of PMOEAm observed at 3.27 ppm at 20 °C decreased considerably at 30 °C and disappeared completely at 50 °C. A similar thermal phase transition was observed in the ^1^H NMR spectra of PMOEAm_50_-*s*-PEOEAm_50_ with a *T*_cp_ of 46.2 °C (Figure S5). However, an increase in *R*_h_ from 16.7 nm at 20 °C to 402.8 nm at 60 °C for PMOEAm_50_-*b*-PEOEAm_50_ and from 18.9 nm at 20 °C to 551.7 nm at 60 °C for PMOEAm_50_-*s*-PEOEm_50_ confirmed the thermal phase transition. In the block and statistical copolymers of PEOEAm and PMOEAm, the small change in *T*_cp_ is due to PEOEAm with a low *T*_cp_ acting as a highly hydrophobic moiety.

## 4. Conclusions

Using the conventional method with SKA^Et^ and the hydrosilylation-promoted method with Me_2_EtSiH, we proceeded in a controlled/living manner to produce PMOEAm and MCIP-PMOEAm, respectively. Using a different initiation method affected only the induction time in the early stages of the polymerization, whereas it had no effect on the propagation rate. The relationship between DP_x_ and *T*_cp_ for PMOEAm_x_ and MCIP-PMOEAm_x_ was very similar, but the *T*_cp_ of MCIP-PMOEAmx was lower than that of PMOEAm_x,_ regardless of the DP_x_ value. Poly(*N*,*N*-disubstituted acrylamide), a polymer segment combined in copolymers of PMOEAm, affected the thermoresponsive properties, with the *T*_cp_ of the block and statistical copolymers consisting of PMOEAm, and nonthermoresponsive and highly water-soluble PDMAm being higher than that of copolymers with thermoresponsive and less water-soluble PEOEAm. For PMOEAm and its block and statistical copolymers, the *T*_cp_ was slightly higher for PMOEAm-*b*-PDMAm than for PMOEAm and considerably higher for PMOEAm-*s*-PDMAm due to the alternating-rich nature of the monomer sequence. Meanwhile, the difference in *T*_cp_ was small between PMOEAm, PMOEAm-*b*-PEOEAm, and PMOEAm-*s*-PEOEAm, and the DP dependence on *T*_cp_ was small for these (co)polymers. These thermoresponsive features are most likely caused by the low *T*_cp_ of PEOEAm. To determine whether a polymer exhibits thermoresponsive properties and the type of comonomers that are appropriate to obtain thermoresponsive copolymers, polymers without substituents at the chain ends should be used. Hydrosilylation-promoted GTP is a reliable tool for resolving these issues and will contribute to the molecular design, synthesis, and application of thermoresponsive polymer materials.

## Figures and Tables

**Table 1:** Synthesis of poly(*N*,*N*-bis(2-methoxyethyl)acrylamide via the hydrosilylation-promoted GTP of MOEAm with Me_2_EtSiH using B(C_6_F_5_)_3_ as the catalyst. *^a^.*

Run	Polymer	[MOEAm]_0_/[Me_2_EtSiH]_0_/[B(C_6_F_5_)_3_]_0_	Time h	*M*_n,calcd_ *^b^* kg mol^−1^	*M*_n,SEC_ (Đ) *^c^* kg mol^−1^	*T*_cp_ *^d^* °C
1	PMOEAm_25_	25/1/0.1	6	4.7	4.5 (1.13)	56.5
2	PMOEAm_50_	50/1/0.1	6	9.4	9.6 (1.16)	53.9
3	PMOEAm_75_	75/1/0.1	12	14.0	13.8 (1.12)	51.2
4	PMOEAm_100_	100/1/0.1	12	18.7	19.0 (1.11)	50.9
5	PMOEAm_150_	150/1/0.2	12	28.1	28.1 (1.12)	50.5
6	PMOEAm_200_	200/1/0.2	18	37.4	37.1 (1.17)	48.0

*^a^* [MOEAm] concentration of 1.0 mol L^−1^ was used in CH_2_Cl_2_ solvent at 25 °C under an Ar atmosphere. Monomer conversion was determined to be >99.9% by ^1^H NMR in CDCl_3_. *^b^* The molecular mass of the polymers was calculated using the equation [MOEAm]/[Me_2_EtSiH]0 × (conv.) × (M.W. of monomer) + (M.W. of H) × 2. *^c^* The molecular mass of polymer was determined by SEC equipped with an RI detector in DMF containing lithium chloride (0.01 mol L^−1^) using PMMA as standards. *^d^* The *T_cp_*s of [MOEAm] was determined by UV–vis measurements in water at 10 g L^−1^.

**Table 2:** Thermoresponsive property and synthesis of PMOEAm-*b*-PDMAm and PMOEAm-*b*-PEOEAm via the hydrosilylation-promoted GTP of MOEAm with Me_2_EtSiH using B(C_6_F_5_)_3_ as the catalyst, followed by the 2nd GTP of DMAm or EOEAm, respectively. *^a^.*

Run	Polymer	1st GTP *^b^*			2nd GTP *^c^*			*T*_cp_ *^e^* °C
		[MOEAm]_0_/[Me_2_EtSiH]_0_	*M*_n,calcd_ /kg mol^−1^	*M*_n,SEC_ (Đ) *^d^* /kg mol^−1^	[DMAm or EOEAm]_0_	*M*_n,calcd_ /kg mol^−1^	*M*_n,SEC_ (Đ) *^d^* /kg mol^−1^	
7	PMOEAm_30_-*b*-PDMAm_70_	30/1	5.6	5.5 (1.09)	70	12.5	12.5 (1.08)	- *^f^*
8	PMOEAm_40_-*b*-PDMAm_60_	40/1	7.5	7.5 (1.10)	60	13.4	13.4 (1.10)	- *^f^*
9	PMOEAm_50_-*b*-PDMAm_50_	50/1	9.4	9.6 (1.13)	50	14.3	14.4 (1.13)	58.0
10	PMOEAm_60_-*b*-PDMAm_40_	60/1	11.2	11.1 (1.10)	40	15.2	15.1 (1.09)	56.5
11	PMOEAm_70_-*b*-PDMAm_30_	70/1	13.1	12.9 (1.11)	30	16.1	16.0 (1.10)	55.8
12	PMOEAm_80_-*b*-PDMAm_20_	80/1	15.0	15.1 (1.12)	20	16.9	16.8 (1.10)	53.3
13	PMOEAm_90_-*b*-PDMAm_10_	90/1	16.8	16.7 (1.13)	10	17.8	17.6 (1.15)	51.4
14	PMOEAm_30_-*b*-PEOEAm_70_	30/1	5.6	5.3 (1.09)	70	20.7	21.0 (1.10)	- *^f^*
15	PMOEAm_40_-*b*-PEOEAm_60_	40/1	7.5	7.2 (1.08)	60	20.4	20.5 (1.09)	- *^f^*
16	PMOEAm_50_-*b*-PEOEAm_50_	50/1	9.4	9.1 (1.12)	50	20.1	20.2 (1.11)	43.5
17	PMOEAm_60_-*b*-PEOEAm_40_	60/1	11.2	10.9 (1.10)	40	19.8	19.4 (1.12)	38.0
18	PMOEAm_70_-*b*-PEOEAm_30_	70/1	13.1	12.8 (1.11)	30	19.6	19.1 (1.10)	33.5
19	PMOEAm_80_-*b*-PEOEAm_20_	80/1	15.0	15.0 (1.11)	20	19.3	19.0 (1.09)	29.7
20	PMOEAm_90_-*b*-PEOEAm_10_	90/1	16.8	16.4 (1.10)	10	19.0	18.8 (1.11)	25.0

*^a^* Solvent, CH_2_Cl_2_; [Me_2_EtSiH]_0_/[B(C_6_F_5_)_3_]_0_, 0.1; room temperature; Ar atmosphere; monomer conversion determined by ^1^H NMR spectra in CDCl_3_, >99.9%. *^b^* [MOEAm]_0_, 1.0 mol L^−1^; time, 12 h and 18 h (runs 17 and 18). *^c^* [DMAm or EOEAm]_0_ = 100 − [MOEAm]_0_; time, 36 h (runs 7−9), 24 h (runs 10−13), 12 h (runs 14−16), and 6 h (runs 17 and 18). *^d^* The molecular mass of the polymers was determined using size exclusion chromatography equipped with a refractive index detector in dimethylformamide (DMF) containing 0.01 mol L^−1^ of lithium chloride and using polymethylmethacrylate (PMMA) as standards. *^e^* The cloud point temperature of [MOEAm] was determined by measuring its ultraviolet-visible spectrum in water at a concentration of 10 g L^−1^. *^f^* Not determined due to insoluble in water.

**Table 3:** Thermoresponsive property of PMOEAm-*s*-PDMAm and PMOEAm-*s*-PEOEAm prepared by the random hydrosilylation-promoted GTcoPs of MOEAm and DMAm or EOEAm with Me_2_EtSiH using B(C_6_F_5_)_3_ as the catalyst, respectively. *^a^.*

Run	Polymer	[MOEAm + DMAm or EOEAm]_0_/[Me_2_EtSiH]_0_	*M*_n,calcd_ kg mol^−1^	*M*_n,SEC_ (Đ) *^b^* kg mol^−1^	*T*_cp_ *^c^* °C
21	PMOEAm_30_-*s*-PDMAm_70_	(30 + 70)/1	12.5	12.5 (1.09)	- *^d^*
22	PMOEAm_40_-*s*-PDMAm_60_	(40 + 60)/1	13.4	13.6 (1.12)	73.4
23	PMOEAm_50_-*s*-PDMAm_50_	(50 + 50)/1	14.3	14.5 (1.14)	72.1
24	PMOEAm_60_-*s*-PDMAm_40_	(60 + 40)/1	15.2	15.3 (1.11)	67.5
25	PMOEAm_70_-*s*-PDMAm_30_	(70 + 30)/1	16.1	16.4 (1.15)	64.2
26	PMOEAm_80_-*s*-PDMAm_20_	(80 + 20)/1	16.9	16.6 (1.07)	59.3
27	PMOEAm_90_-*s*-PDMAm_10_	(90 + 10)/1	17.8	17.7 (1.11)	55.5
28	PMOEAm_30_-*s*-PEOEAm_70_	(30 + 70)/1	20.7	20.9 (1.15)	- *^e^*
29	PMOEAm_40_-*s*-PEOEAm_60_	(40 + 60)/1	20.4	20.6 (1.14)	- *^e^*
30	PMOEAm_50_-*s*-PEOEAm_50_	(50 + 50)/1	20.1	20.5 (1.13)	46.2
31	PMOEAm_60_-*s*-PEOEAm_40_	(60 + 40)/1	19.8	19.3 (1.09)	45.3
32	PMOEAm_70_-*s*-PEOEAm_30_	(70 + 30)/1	19.6	19.0 (1.10)	42.4
33	PMOEAm_80_-*s*-PEOEAm_20_	(80 + 20)/1	19.3	18.9 (1.12)	37.0
34	PMOEAm_90_-*s*-PEOEAm_10_	(90 + 10)/1	19.0	18.6 (1.12)	33.7

*^a^* Solvent, CH_2_Cl_2_; [MOEAm + DMAm or EOEAm]_0_, 1.0 mol L^−1^; [Me_2_EtSiH]_0_/[B(C_6_F_5_)_3_]_0_, 1; temp., 25 °C; time, 24 h; Ar atmosphere; monomer conversion determined by ^1^H NMR spectra in CDCl_3_, >99.9%. *^b^* The molecular mass of the polymers was determined using size exclusion chromatography equipped with a refractive index detector in dimethylformamide (DMF) containing 0.01 mol L^−1^ of lithium chloride and using polymethylmethacrylate (PMMA) as standards.*^c^* Determined by UV–vis measurements in water (10 g L^−1^). *^d^* Water-soluble but no *T*_cp_ under 95 °C. *^e^* Not determined due to insoluble in water.

**Table 4:** *R*_h_ values for PMOEAm_50_, MCIP-PMOEAm_50_, PMOEAm_50_-*b*-PDMAm_50_, PMOEAm_50_-*s*-PDMAm_50_, PMOEAm_50_-*b*-PEOEAm_50_, and PMOEAm_50_-*s*-PEOEAm_50_ at 20 °C and 60 °C.

Polymer Code	*T*_cp_ *^a^*	*R*_h_ *^b^*/nm	
		20 °C	60 °C
PMOEAm_50_	53.9	15.2	425.6
MCIP-PMOEAm_50_	40.8	16.1	457.2
PMOEAm_50_-*b*-PDMAm_50_	58.0	16.7	402.8
PMOEAm_50_-*s*-PDMAm_50_	72.1	14.5	349.3 *^c^*
PMOEAm_50_-*b*-PEOEAm_50_	25.0	18.9	551.7
PMOEAm_50_-*s*-PEOEAm_50_	33.7	18.3	482.6

*^a^* The cloud point temperature of [MOEAm] was determined by measuring its ultraviolet-visible spectrum in water at a concentration of 10 g L^−1^. ^*b*^ Determined by DLS measurements in 10 g L^−1^ water by dynamic light scattering (DLS). *^c^* At 75 °C.

## Data Availability

Data are contained within the article and supplementary materials.

## Supplementary Materials

The following supporting information can be downloaded at https://www.mdpi.com/article/10.3390/polym15244681/s1: Table S1. Synthesis of MCIP-PMOEAm by GTP of MOEAm with SKA^Et^ using B(C_6_F_5_)_3_ as the catalyst.; Table S2. Random group transfer copolymerization (GTcoP) of MOEAm and DMAm with Me_2_EtSiH using B(C_6_F_5_)_3_ as the catalyst.; Table S3. Random group transfer copolymerization (GTcoP) of MOEAm and EOEAm with Me_2_EtSiH using B(C_6_F_5_)_3_ as the catalyst.; Figure S1. SEC traces of PMOEAm_x_-*b*-PDMAm_y_ with (a) x/y = 30/70, (b) x/y = 40/60, (c) x/y = 50/50, (d) x/y = 60/40, (e) x/y = 70/30, (f) x/y = 80/20, and (g) x/y = 90/10 and PMOEAm_x_-*b*-PEOEAm_y_ with (h) x/y = 50/50, (i) x/y = 60/40, (j) x/y = 70/30, (k) x/y = 80/20, and (l) x/y = 90/10 (eluent, DMF containing lithium chloride (0.01 mol L^−1^); flow rate, 1.0 mL min^−1^).; Figure S2. SEC traces of PMOEAm_x_-*s*-PDMAm_y_ with (a) x/y = 30/70, (b) x/y = 40/60, (c) x/y = 50/50, (d) x/y = 60/40, (e) x/y = 70/30, (f) x/y = 80/20, and (g) x/y = 90/10 and PMOEAm_x_-*s*-PEOEAm_y_ with (h) x/y = 50/50, (i) x/y = 60/40, (j) x/y = 70/30, (k) x/y = 80/20, and (l) x/y = 90/10 (eluent, DMF containing lithium chloride (0.01 mol L^−1^); flow rate, 1.0 mL min^−1^).; Figure S3. ^1^H NMR spectra of (a) PMOEAm_50_-*b*-PDMAm_50_ and (b) PMOEAm_50_-*s*-PDMAm_50_ in CDCl_3_.; Figure S4. ^1^H NMR spectra of (a) PMOEAm_50_-*b*-PEOEAm_50_ and (b) PMOEAm_50_-*s*-PEOEAm_50_ in CDCl_3_.; Figure S5. ^1^H NMR spectra of PMOEAm_50_-*s*-PDMAm_50_ measured at 20, 30, and 50 °C in D_2_O.; Figure S6. ^1^H NMR spectra of PMOEAm_50_-*s*-PEOEAm_50_ measured at 20, 35, and 50 °C in D_2_O.; Figure S7. UV-vis absorption spectra of (a) PMOEAm, (b) MCIP-PMOEAm, (c) PMOEAm-*s*-PDMAm, (d) PMOEAm-*b*-PDMAm, (e) PMOEAm-s-PEOEAm, and (f) PMOEAm-b-PEOEAm in water (10 g L^−1^) at different temperatures.; Figure S8. *R*_h_ values for (a) PMOEAm_50_, (b) MCIP-PMOEAm_50_, (c) PMOEAm_50_-*s*-PDMAm_50_, (d) PMOEAm_50_-*b*-PDMAm_50_, (e) PMOEAm_50_-*s*-PEOEAm_50_, and (f) PMOEAm_50_-*b*-PEOEAm_50_ at 20 and 60 °C.
