# Supplementary material for: Thermoresponsive Property of Poly(N,N-bis(2-methoxyethyl)acrylamide) and Its Copolymers with Water-Soluble Poly(N,N-disubstituted acrylamide) Prepared Using Hydrosilylation-Promoted Group Transfer Polymerization
Source: Polymers (Basel). 2023 Dec 12;15(24):4681. doi: 10.3390/polym15244681 (PMC10747282; doi:10.3390/polym15244681)
Supplement: Supplementary file 1 [file polymers-15-04681-s001.zip › polymers-2727343-supplementary.pdf]

Supporting Information

Thermoresponsive Property of Poly(*N*,*N*-bis(2-methoxyethyl)acrylamide) Its Copolymers with Water-Soluble Poly(*N*,*N*-disubstituted acrylamide) Prepared Using Hydrosilylation-Promoted Group Transfer Polymerization

Xiangming Fu,[a] Yanqiu Wang,[a] Liang Xu,[a] Atsushi Narumi,[b] Shin-ichiro Sato,[c] Xiaoran Yang,[a] Xiande Shen,*[a,d] and Toyoji Kakuchi*[a,c,d]

[a] Research Center for Polymer Materials, School of Materials Science and Engineering, Changchun University of Science and Technology, Weixing Road 7989, Jilin 130022, China

[b] Graduate School of Organic Materials Science, Yamagata University, 4-3-16 Jonan, Yonezawa, Yamagata 992-8510, Japan

[c] Division of Applied Chemistry, Faculty of Engineering, Faculty of Engineering, Hokkaido University, Sapporo, Hokkaido 060-8628, Japan

[d] Chongqing Research Institute, Changchun University of Science and Technology, No. 618 Liangjiang Avenue, Longxing Town, Yubei District, Chongqing City 401135, China

CORRESPONDING AUTHOR FOOTNOTE

Tel & Fax: +81-11-706-6602. E-mail: kakuchi@eng.hokudai.ac.jp

1. Synthesis of MCIP-PMOEAm.

Table S1. Synthesis of MCIP-PMOEAm by GTP of MOEAm with SKA^{Et} using $B(C_6F_5)_3$ as the catalyst. I would like to cancel above experiment.

run	Polymer code	$[MOEAm]_0/[SKA^{Et}]_0/[B(C_6F_5)_3]_0$	$M_{n,calcd}$ [b] kg mol^{-1}	M_n (M_w/M_n) [c] kg mol^{-1}	T_{cp} [d]
1	MCIP-$PMOEAm_{25}$	25/1/0.1	4.9	5.1 (1.11)	41.5
2	MCIP-$PMOEAm_{50}$	50/1/0.1	9.6	10.2 (1.13)	40.8
3	MCIP-$PMOEAm_{75}$	75/1/0.1	14.2	14.4 (1.12)	39.7
4	MCIP-$PMOEAm_{100}$	100/1/0.1	18.9	18.6 (1.15)	38.0
5	MCIP-$PMOEAm_{150}$	150/1/0.2	28.3	27.9 (1.14)	36.2
6	MCIP-$PMOEAm_{200}$	200/1/0.2	37.6	38.8 (1.15)	34.5

[a] $[MOEAm]_0$, 1.0 mol L^{-1}; solvent, CH_2Cl_2; temp., 25 °C; Ar atmosphere; monomer conversion determined by ^{1}H NMR in $CDCl_3$, >99.9%, time, 8 h for runs 1−4 and 12 h for runs 5 and 6. [b] Calculated using the equation of $[MOEAm]_0/[SKA^{Et}]_0$ x (conv.) x (MW of MOEAm) + (MW of $(CH_3OCOC(CH_3)_2$ + H)). [c] Determined by SEC equipped with a RI detector in DMF containing lithium chloride (0.01 mol L^{-1}) using PMMA standards. [d] Determined by UV-vis measurements in water (10 g L^{-1}).

2. Synthesis of PEOEAm.

The hydrosilylation-promoted GTP of EOEAm with Me_2EtSiH using $B(C_6F_5)_3$ as the catalyst synthesis of PEOEAm performed to prepare the $PEOEAm_x$ with $M_{n,SEC}$ (M_w/M_n) and T_{cp} as follows. x = 25, 5.4 kg mol^{-1} (1.15) and 14.5 °C; x = 50, 10.5 kg mol^{-1} (1.15) and 13.9 °C; x = 15.9 kg mol^{-1} (1.12) and 8.9 °C; x = 100, 21.8 kg mol^{-1} (1.11) and 8.0 °C; x = 150, 31.8 kg mol^{-1} (1.10) and 5.9 °C; x = 200, 42.5 kg mol^{-1} (1.10) and 5.0 °C.

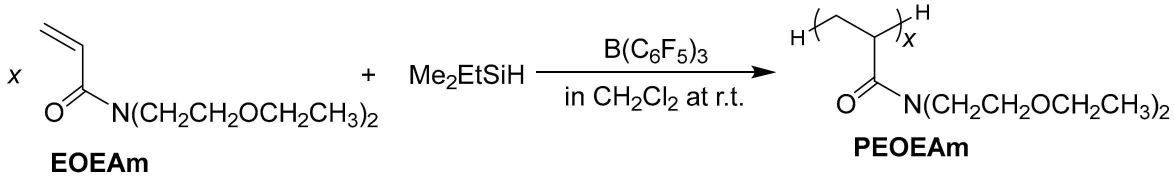

3. Monomer reactivity ratio

Random GTcoP of MOEAm and DMAm. The monomer reactivity ratios of r_{MOEAm} and r_{DMAm} were determined using the Kelen–Tüdös equation of $\eta = (r_{\mathrm{MOEAm}} + r_{\mathrm{DMAm}}/\alpha)\xi - r_{\mathrm{DMAm}}/\alpha)$, where η and ξ are mathematical functions of the comonomer molar fractions in the feed and in the copolymer, respectively: $\eta = G/(\alpha + F)$ and $\xi = F/(\alpha + F)$. Here, $G = x(y - 1)/y$ and $F = x^2/y$ with $x = M_{\mathrm{MOEAm}}/M_{\mathrm{DMAm}}$ and $y = m_{\mathrm{MOEAm}}/m_{\mathrm{DMAm}}$. F_{MOEAm} and F_{DMAm} are the mole fractions of MOEAm and DMAm in the monomer feed, respectively, M_{MOEAm} and M_{DMAm} are the mole fractions of MOEAm and DMAm in the residual monomer mixture, respectively, and m_{MOEAm} and m_{DMAm} are the mole fractions of MOEAm and DMAm units in the copolymer, respectively. The term $\alpha = \sqrt{F_m F_{\mathrm{M}}}$ is a constant that is chosen appropriately to obtain a uniform spread of the data ($\alpha > 0$). F_{m} and F_{M} are the lowest and highest values obtained from the experimental data. The monomer reactivity ratios r_{MOEAm} and r_{DMAm} were determined to be 0.39 and 0.94, respectively, from the least-squares method. The number-average sequence length of the MOEAm unit (l_{MOEAm}) was determined as a parameter that reflects the isolation tendency of the MOEAm–MOEAm diad. The $l_{\mathrm{MOEAm}n}$ was calculated from the equation of $l_{\mathrm{MOEAm}} = 1 + r_{\mathrm{MOEAm}}[F_{\mathrm{MOEAm}}/(1 - F_{\mathrm{MOEAm}})]$. In addition, he $l_{\mathrm{DMAm}n}$ was calculated from the equation of $l_{\mathrm{DMAm}} = 1 + r_{\mathrm{DMAm}}[F_{\mathrm{DMAm}}/(1 - F_{\mathrm{DMAm}})]$.

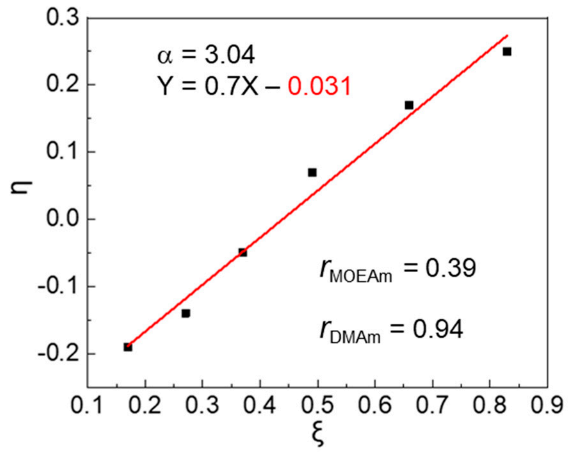

Table S2. Random group transfer copolymerization (GTcoP) of MOEAm and DMAm with Me_2EtSiH using $B(C_6F_5)_3$ as the catalyst [a]

$F_{MOEAm} + F_{DMAm} = 1$		$M_{MOEAm} + M_{DMAm} = 1$ [b]		conv. %	$m_{MOEAm} + m_{DMAm} = 1$ [b]		ξ	η	l_{MOEAm}	l_{DMAm}
F_{MOEAm}	F_{DMAm}	M_{MOEAm}	M_{DMAm}		m_{MOEAm}	m_{DMAm}				
0.3	0.7	0.33	0.67	0.17	0.28	0.72	0.17	-0.19	1.17	3.19
0.4	0.6	0.45	0.55	0.18	0.37	0.63	0.27	-0.14	1.26	2.41
0.5	0.5	0.55	0.45	0.19	0.46	0.54	0.37	-0.05	1.39	1.94
0.6	0.4	0.66	0.34	0.20	0.56	0.44	0.49	0.07	1.59	1.63
0.7	0.3	0.77	0.23	0.21	0.65	0.35	0.66	0.17	1.91	1.40
0.8	0.2	0.87	0.13	0.18	0.75	0.25	0.83	0.25	2.56	1.27

[a] $[Me_2EtSiH]_0/[B(C_6F_5)_3]_0 = 1/0.1$; solvent, CH_2Cl_2; temperature, 25 °C. [b] Determined by ^1H NMR spectroscopy in $CDCl_3$.

Random GTcoP of MOEAm and EOEAm. The monomer reactivity ratios of r_{MOEAm} and r_{EOEAm} were 1.45 and 1.03, respectively, which were determined using the same method for determining r_{MOEAm} and r_{DMAm}. In addition, the number-average sequence lengths of the MOEAm unit (l_{MOEAm}) and EOEAm unit (l_{EOEAm}) were calculated using $l_{MOEAm} = 1 + r_{MOEAm}[F_{MOEAm}/(1 - F_{MOEAm})]$ and $l_{DMAm} = 1 + r_{DMAm}[F_{DMAm}/(1 - F_{DMAm})]$, respectively.

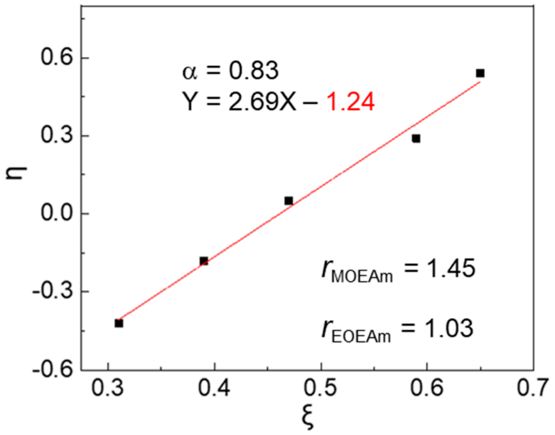

Table S3. Random group transfer copolymerization (GTcoP) of MOEAm and EOEAm with Me_2EtSiH using $B(C_6F_5)_3$ as the catalyst [a]

$F_{MOEAm} + F_{EOEAm} = 1$		$M_{MOEAm} + M_{EOEAm} = 1$ [b]		conv. %	$m_{MOEAm} + m_{EOEAm} = 1$ [b]		ξ	η	l_{MOEAm}	l_{EOEAm}
F_{MOEAm}	F_{EOEAm}	M_{MOEAm}	M_{EOEAm}		m_{MOEAm}	m_{EOEAm}				
0.3	0.7	0.29	0.71	0.20	0.31	0.69	0.31	-0.42	1.62	3.40
0.4	0.6	0.38	0.62	0.21	0.42	0.48	0.39	-0.18	1.97	2.55
0.5	0.5	0.47	0.53	0.19	0.52	0.52	0.47	0.05	2.45	2.03
0.6	0.4	0.58	0.42	0.20	0.62	0.38	0.59	0.27	3.18	1.69
0.7	0.3	0.67	0.33	0.20	0.73	0.73	0.65	0.54	4.38	1.44
0.8	0.2	0.76	0.24	0.22	0.84	0.16	0.70	0.85	6.80	1.26

[a] $[Me_2EtSiH]_0/[B(C_6F_5)_3]_0 = 1/0.1$; solvent, CH_2Cl_2; temperature, 25 °C. [b] Determined by ^1H NMR spectroscopy in $CDCl_3$.

4. SEC traces of PMOEAm and its block and statistical copolymers.

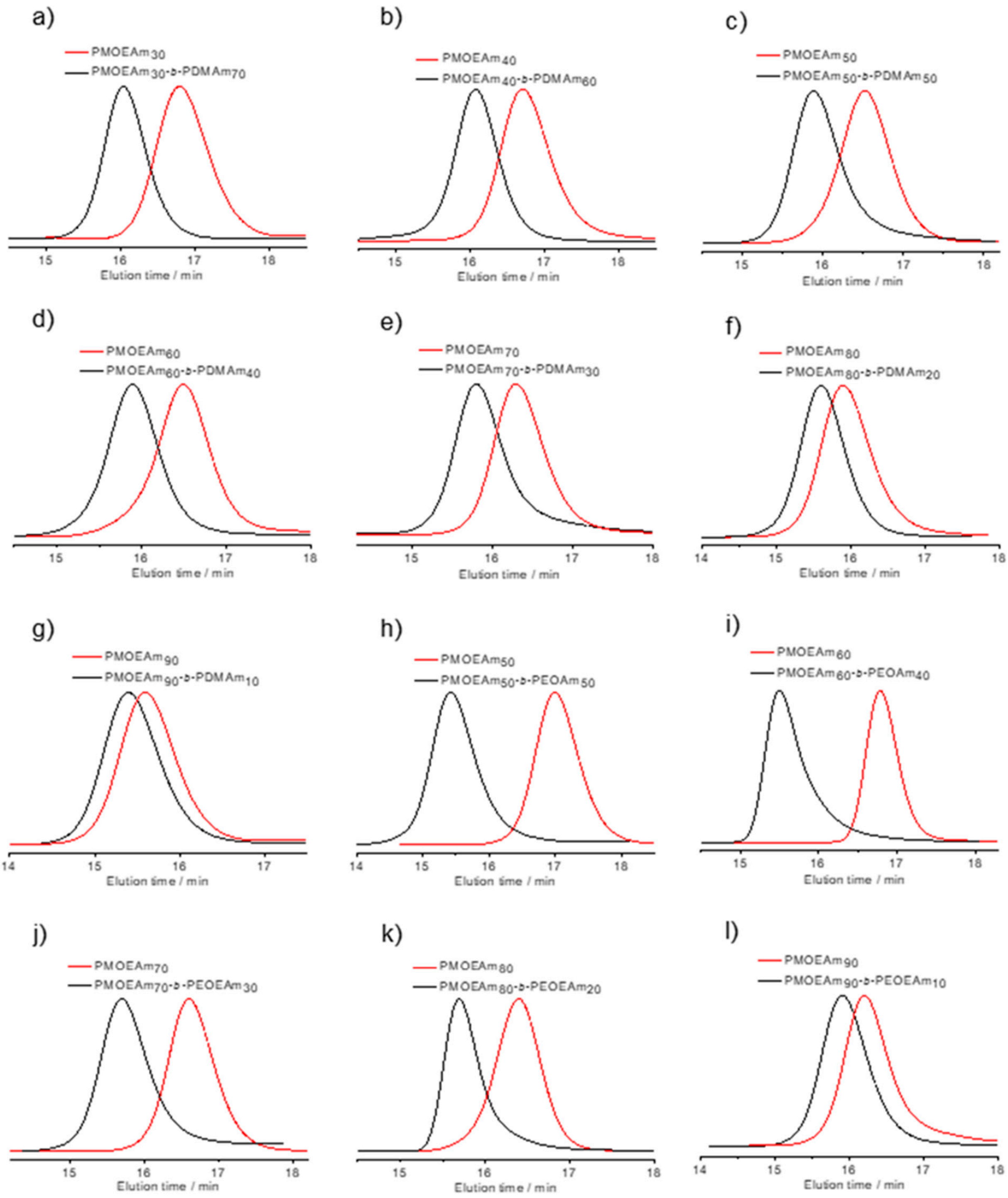

Figure S1. SEC traces of PMOEAm$_x$-*b*-PDMAm$_y$ with a) x/y = 30/70, b) x/y = 40/60, c) x/y = 50/50, d) x/y = 60/40, e) x/y = 70/30, f) x/y = 80/20, and g) x/y = 90/10 and PMOEAm$_x$-*b*-PEOEAm$_y$ with h) x/y = 50/50, i) x/y = 60/40, j) x/y = 70/30, k) x/y = 80/20, and l) x/y = 90/10 (eluent, DMF containing lithium chloride (0.01 mol L^{-1}); flow rate, 1.0 mL min^{-1}).

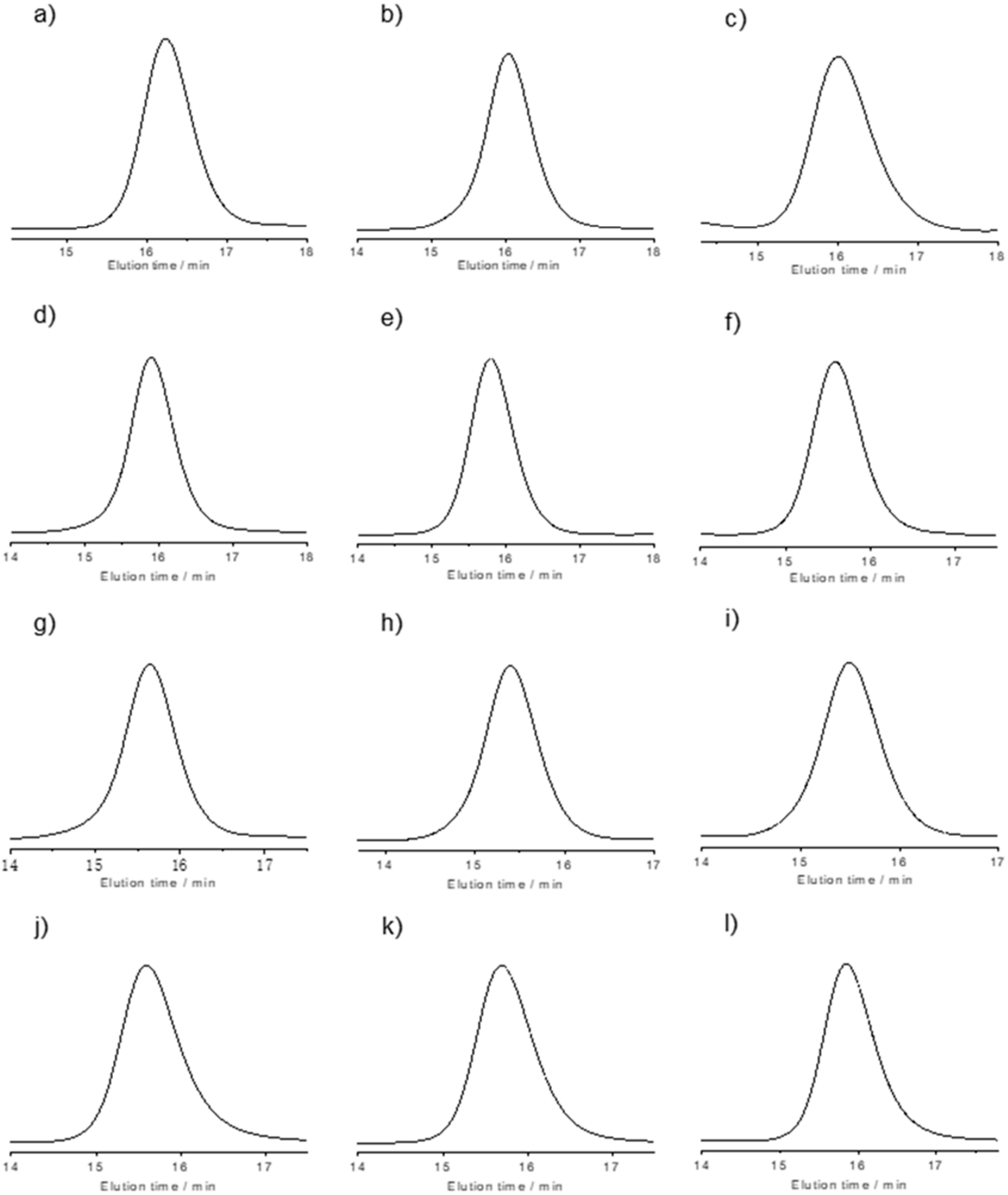

Figure S2. SEC traces of PMOEAm$_x$-*s*-PDMAm$_y$ with a) x/y = 30/70, b) x/y = 40/60, c) x/y = 50/50, d) x/y = 60/40, e) x/y = 70/30, f) x/y = 80/20, and g) x/y = 90/10 and PMOEAm$_x$-*s*-PEOEAm$_y$ with h) x/y = 50/50, i) x/y = 60/40, j) x/y = 70/30, k) x/y = 80/20, and l) x/y = 90/10 (eluent, DMF containing lithium chloride (0.01 mol L^{-1}); flow rate, 1.0 mL min^{-1}).

5. ^{1}H NMR spectra of bock copolymers

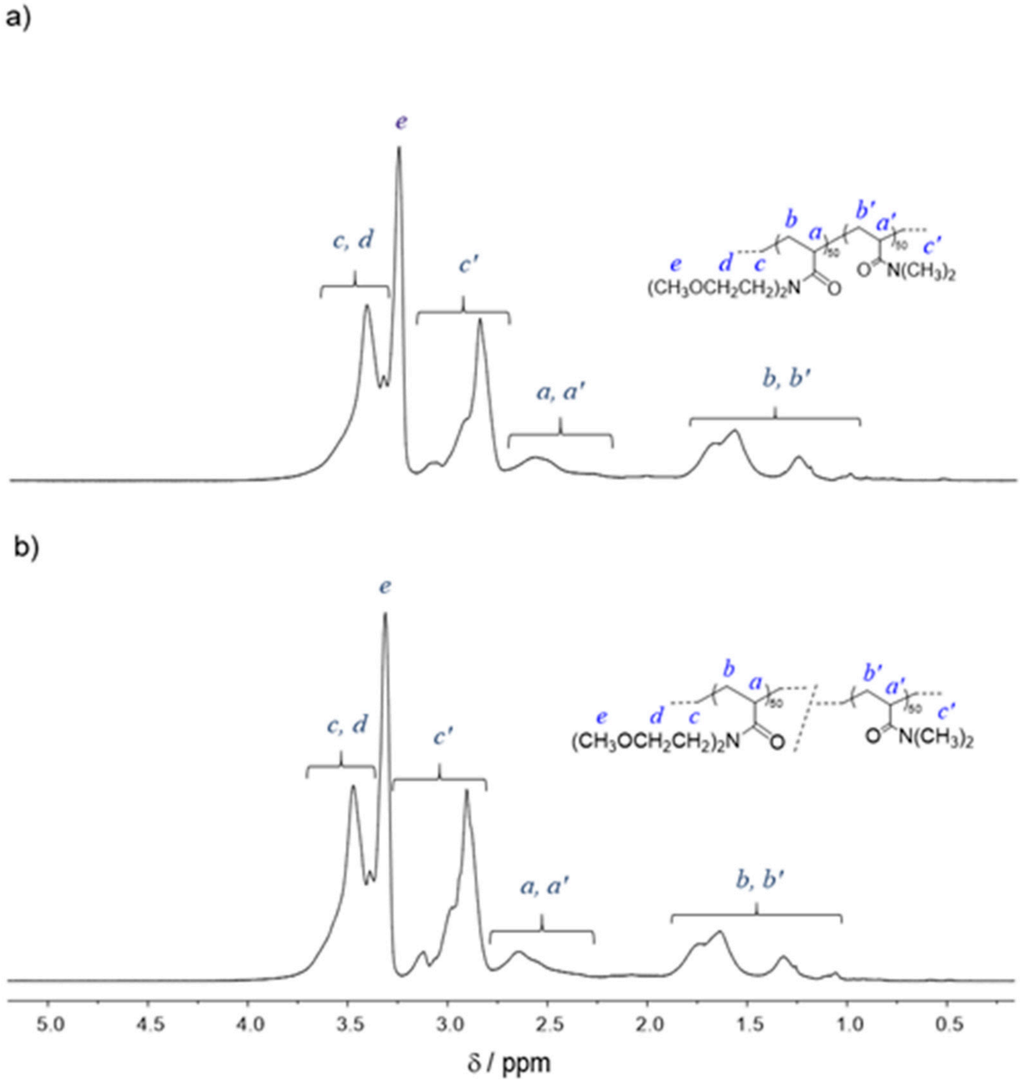

Figure S3. ^{1}H NMR spectra of a) PMOEAm$_{50}$-*b*-PDMAm$_{50}$ and b) PMOEAm$_{50}$-*s*-PDMAm$_{50}$ in $CDCl_3$.

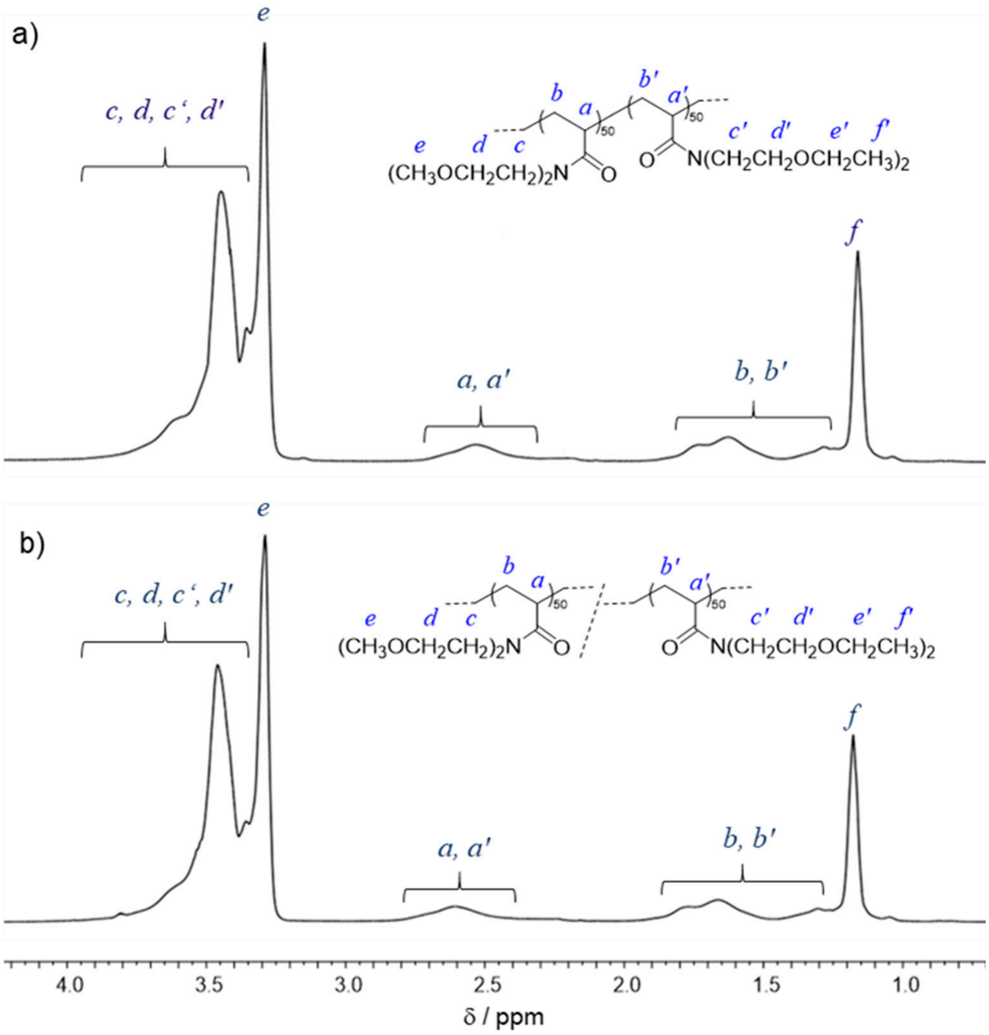

Figure S4. ^{1}H NMR spectra of a) $PMOEAm_{50}$-*b*-$PEOEAm_{50}$ and b) $PMOEAm_{50}$-*s*-$PEOEAm_{50}$ in $CDCl_3$.

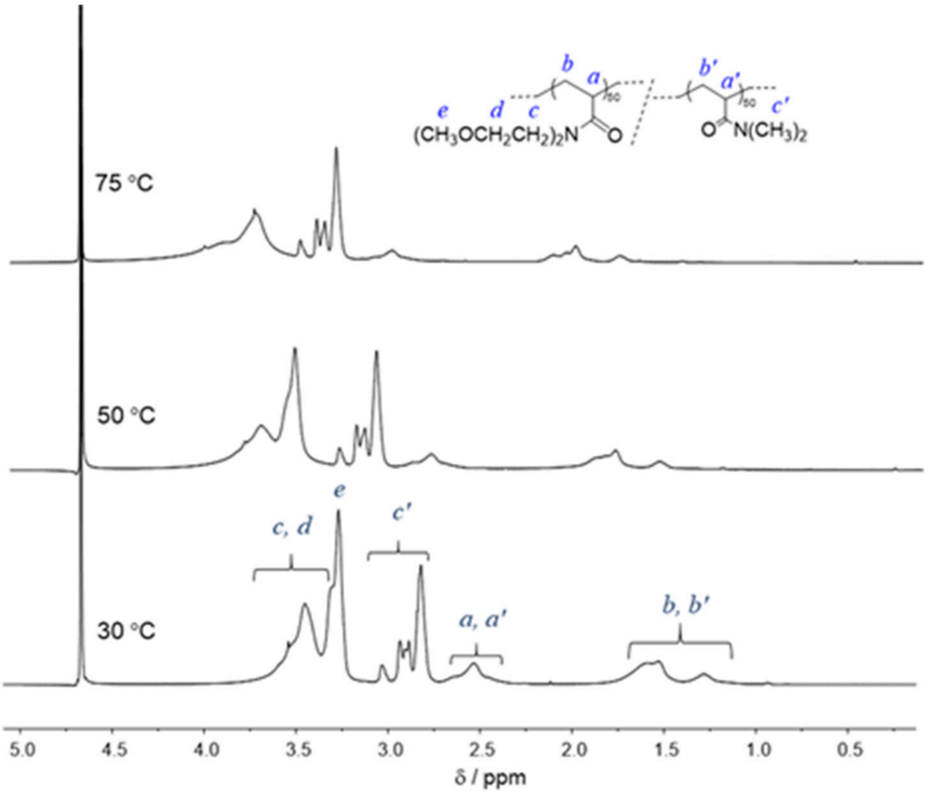

Figure S5. ^{1}H NMR spectra of $PMOEAm_{50}$-*s*-$PDMAm_{50}$ measured at 20, 30, and 50 °C in D_2O.

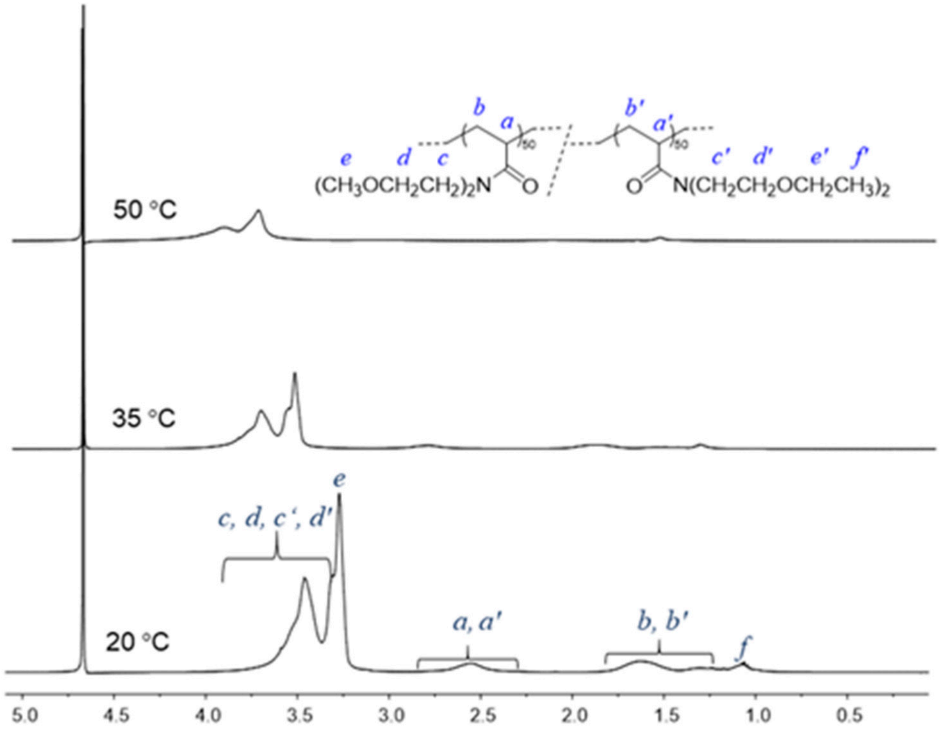

Figure S6. ^{1}H NMR spectra of PMOEAm$_{50}$-*s*-PEOEAm$_{50}$ measured at 20, 35, and 50 °C in D_2O.

6. Determination of cloud-point temperature (T_{cp}) by UV-vis spectrophotometry

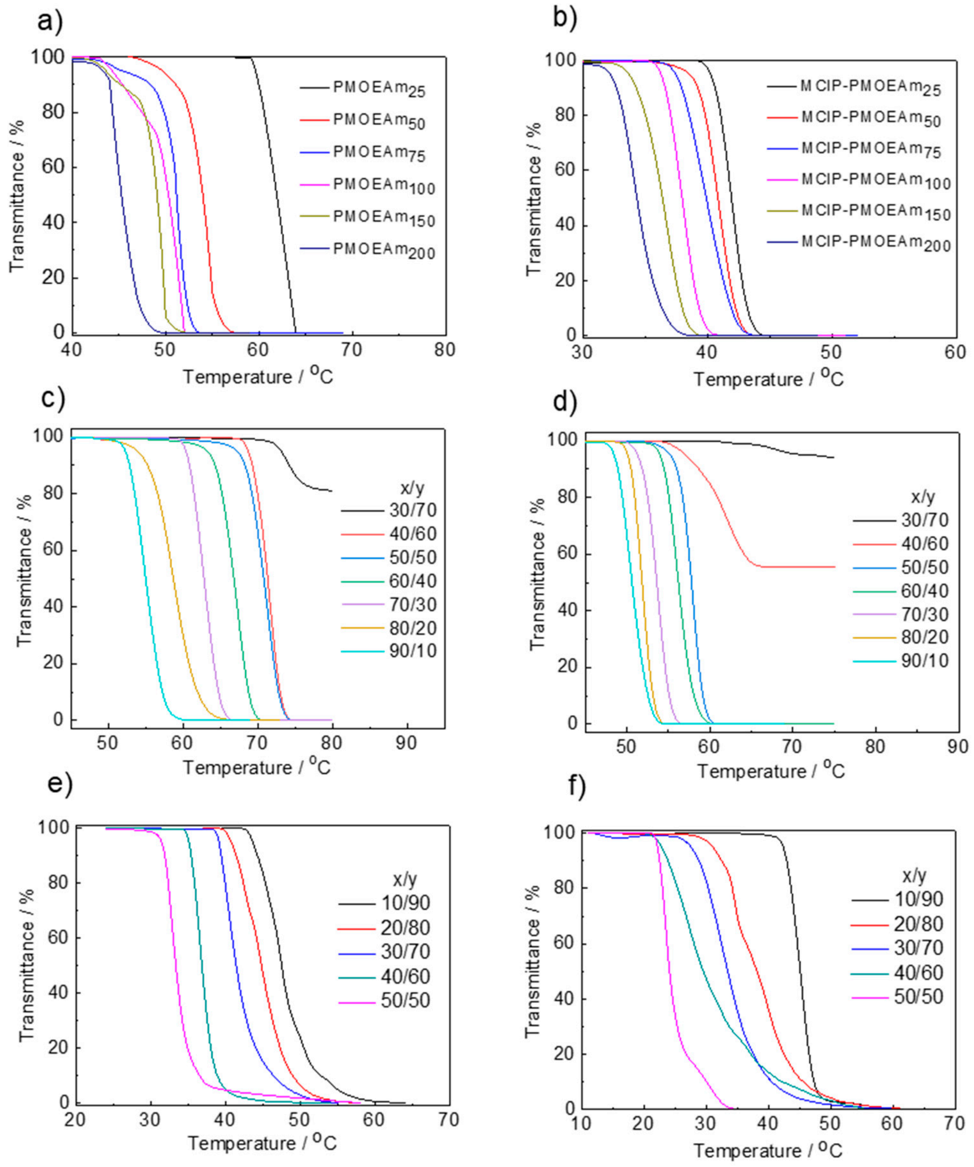

Figure S7. UV-vis absorption spectra of a) PMEAm, b) MCIP-PMOEAm, c) PMOEAm-*s*-PDMAm, d) PMOEAm-*b*-PDMAm, e) PMOEAm-s-PEOEAm and f) PMOEAm-b-PEOEAm in water (10 g L^{-1}) at different temperatures.

7. Dynamic light scattering (DLS) measurement of PMOEAm and its copolymers

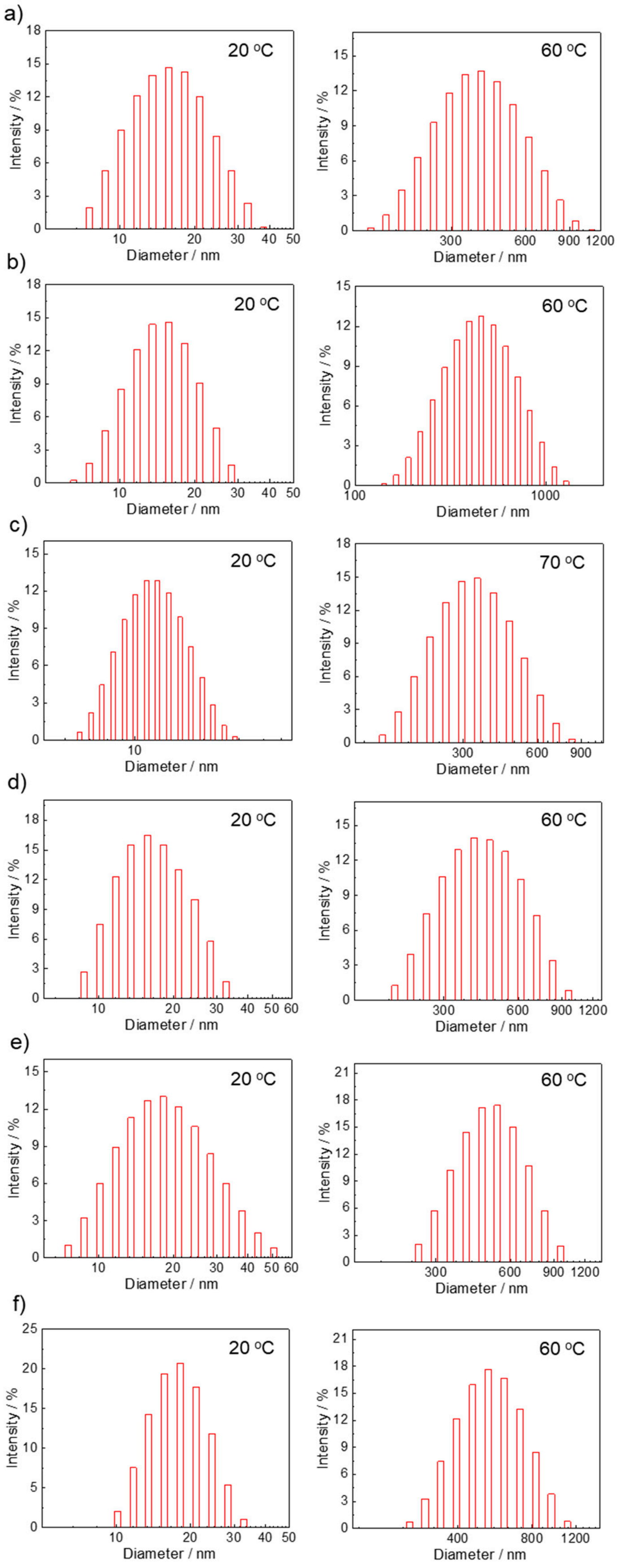

Figure S8. R_h values for a) $PMOEAm_{50}$, b) MCIP-$PMOEAm_{50}$, c) $PMOEAm_{50}$-*s*-$PDMAm_{50}$, d) $PMOEAm_{50}$-*b*-$PDMAm_{50}$, e) $PMOEAm_{50}$-*s*-$PEOEAm_{50}$, and f) $PMOEAm_{50}$-*b*-$PEOEAm_{50}$ at 20 and 60 °C.
